# Electronic Health Record–Embedded Individualized Pain Plans for Emergency Department Treatment of Vaso-occlusive Episodes in Adults With Sickle Cell Disease: Protocol for a Preimplementation and Postimplementation Study

**DOI:** 10.2196/24818

**Published:** 2021-04-16

**Authors:** Lingzi Luo, Allison A King, Yvonne Carroll, Ana A Baumann, Donald Brambilla, Christopher R Carpenter, Joseph Colla, Robert W Gibson, Siera Gollan, Greg Hall, Lisa Klesges, Abdullah Kutlar, Matthew Lyon, Cathy L Melvin, Sarah Norell, Martina Mueller, Michael B Potter, Rachel Richesson, Lynne D Richardson, Gery Ryan, Lauren Siewny, Marsha Treadwell, Leslie Zun, Janelle Armstrong-Brown, Lisa Cox, Paula Tanabe

**Affiliations:** 1 Washington University School of Medicine St Louis, MO United States; 2 St Jude Children’s Research Hospital Memphis, TN United States; 3 Washington University in St. Louis St Louis, MO United States; 4 RTI International Durham, NC United States; 5 University of Illinois Chicago Chicago, IL United States; 6 Augusta University Augusta, GA United States; 7 Medical University of South Carolina Charleston, SC United States; 8 University of Memphis Memphis, TN United States; 9 University of California San Francisco San Francisco, CA United States; 10 Duke University Durham, NC United States; 11 Icahn School of Medicine at Mount Sinai New York, NY United States; 12 The RAND Corporation Santa Monica, CA United States; 13 Chicago Medical School North Chicago, IL United States

**Keywords:** sickle cell disease, RE-AIM, emergency department care, pain treatment, digital medicine, health innovation, implementation science, patient portal, electronic health record

## Abstract

**Background:**

Individuals living with sickle cell disease often require aggressive treatment of pain associated with vaso-occlusive episodes in the emergency department. Frequently, pain relief is poor. The 2014 National Heart, Lung, and Blood Institute evidence-based guidelines recommended an individualized treatment and monitoring protocol to improve pain management of vaso-occlusive episodes.

**Objective:**

This study will implement an electronic health record–embedded individualized pain plan with provider and patient access in the emergency departments of 8 US academic centers to improve pain treatment for adult patients with sickle cell disease. This study will assess the overall effects of electronic health record–embedded individualized pain plans on improving patient and provider outcomes associated with pain treatment in the emergency department setting and explore barriers and facilitators to the implementation process.

**Methods:**

A preimplementation and postimplementation study is being conducted by all 8 sites that are members of the National Heart, Lung, and Blood Institute–funded Sickle Cell Disease Implementation Consortium. Adults with sickle cell disease aged 18 to 45 years who had a visit to a participating emergency department for vaso-occlusive episodes within 90 days prior to enrollment will be eligible for inclusion. Patients will be enrolled in the clinic or remotely. The target analytical sample size of this study is 160 patient participants (20 per site) who have had an emergency department visit for vaso-occlusive episode treatment at participating emergency departments during the study period. Each site is expected to enroll approximately 40 participants to reach the analytical sample size. The electronic health record–embedded individualized pain plans will be written by the patient’s sickle cell disease provider, and sites will work with the local informatics team to identify the best method to build the electronic health record–embedded individualized pain plan with patient and provider access. Each site will adopt required patient and provider implementation strategies and can choose to adopt optional strategies to improve the uptake and sustainability of the intervention. The study is informed by the Technology Acceptance Model 2 and the Reach, Effectiveness, Adoption, Implementation, and Maintenance framework. Provider and patient baseline survey, follow-up survey within 96 hours of an emergency department vaso-occlusive episode visit, and selected qualitative interviews within 2 weeks of an emergency department visit will be performed to assess the primary outcome, patient-perceived quality of emergency department pain treatment, and additional implementation and intervention outcomes. Electronic health record data will be used to analyze individualized pain plan adherence and additional secondary outcomes, such as hospital admission and readmission rates.

**Results:**

The study is currently enrolling study participants. The active implementation period is 18 months.

**Conclusions:**

This study proposes a structured, framework-informed approach to implement electronic health record–embedded individualized pain plans with both patient and provider access in routine emergency department practice. The results of the study will inform the implementation of electronic health record–embedded individualized pain plans at a larger scale outside of Sickle Cell Disease Implementation Consortium centers.

**Trial Registration:**

ClinicalTrials.gov NCT04584528; https://clinicaltrials.gov/ct2/show/NCT04584528.

**International Registered Report Identifier (IRRID):**

DERR1-10.2196/24818

## Introduction

### Background

Sickle cell disease is an inherited red blood cell disorder affecting approximately 100,000 people in the United States, predominantly African Americans [1]. In the past few decades, clinical interventions have facilitated significant improvement in patient outcomes. The survival rate to adulthood in sickle cell disease improved from less than 50% in 1970 [2] to nearly 95% in 2010 [3], and the median age at death of patients with sickle cell disease increased from 28 years in 1979 to 43 years in 2014 [4].

Individuals with sickle cell disease often experience acute painful events—vaso-occlusive episodes—during which the transfer of oxygen and nutrients to tissues is decreased because of blood vessel blockage from polymerization [5]. These episodes are characterized by sudden onset of excruciating pain, often requiring high doses of opioids. Historically, pain treatment in the emergency department for individuals with sickle cell disease has been challenging [6]. There is a growing demand to improve the treatment of vaso-occlusive episodes in adults with sickle cell disease, especially in emergency departments, where patients with sickle cell disease require immediate pain treatment [7].

In 2014, the National Heart, Lung, and Blood Institute published an expert panel report [8] of evidence-based recommendations for sickle cell disease management. The use of “an individualized prescribing and monitoring protocol or an SCD-specific protocol whenever possible” [8] in all settings was among the treatment recommendations for vaso-occlusive episode treatment. For pediatric patients with sickle cell disease, studies have found that those treated with individualized pain plans (IPPs) had fewer hospital admissions and readmissions as well as improved pain scores [9-11]. In these studies, providers perceived that having an IPP improved the efficiency and quality of pain management [9,11]. For adult patients with sickle cell disease, however, the literature on the use of IPPs is scarce. A randomized controlled trial has shown that adult patients randomized to patient-specific or weight-based opioid pain plans in the electronic health record (EHR) experienced a significantly greater reduction in pain scores from emergency department arrival to emergency department discharge and a lower hospital admission rate [12]. Another retrospective study found decreased time to first opioid and length of emergency department stay after IPP implementation [13]. EHR use increased from 9.4% in 2008 to 83.8% in 2015 in nonfederal acute care hospitals [14], so these EHR-embedded individualized pain plans are now possible in most hospitals if planned with collaborative efforts and informed by frameworks from Implementation Science [15].

The Sickle Cell Disease Implementation Consortium was established in 2016 to improve the health and well-being of adolescents and young adults with sickle cell disease [16]. The Sickle Cell Disease Implementation Consortium is a cooperative research program of 8 clinical centers; a data coordinating center; and the National Heart, Lung, and Blood Institute to promote quality of care for patients with sickle cell disease between the ages of 15 and 45 years. Investigators in the Sickle Cell Disease Implementation Consortium conducted a systematic literature review and a comprehensive needs assessment among the 8 participating centers to identify 3 key areas of improvement to address [17]. One of the major opportunities for improvement was the treatment of pain in the adult emergency department.

Patients with sickle cell disease and providers often report frustration with emergency department care, and emergency department providers may have negative attitudes toward individuals living with sickle cell disease [18-21]. These factors may result in patients having lower levels of care satisfaction and the delay or avoidance of emergency department care [17,21,22]. Similarly, from the Sickle Cell Disease Implementation Consortium needs assessment, patients reported being less pleased with their emergency department care compared to their routine care, with only approximately half of participants being satisfied or perceiving adequate quality care in the emergency department [17]. Across 8 sites, 65.7% of the 437 respondents reported that they required emergency department care for acute episodes of pain, and 34.6% of respondents reported 3 or more hospital admissions for pain in the previous year, which is consistent with previous reports in the literature [17]. Slightly fewer than half of the emergency department provider respondents reported that either their emergency department does not have a pain treatment protocol or they were unaware if such a protocol exists [23]. The needs assessment results suggested that establishing standardized treatment in adult emergency departments is key to improved clinical outcomes and care-seeking experiences for adult patients with sickle cell disease across 8 Sickle Cell Disease Implementation Consortium sites. Members from the Sickle Cell Disease Implementation Consortium formed a study workgroup to improve emergency department care for adult patients with sickle cell disease. The workgroup is composed of investigators from the 8 participating Sickle Cell Disease Implementation Consortium sites, representatives from the data coordinating center, and patient, caregiver, and community stakeholders.

### Study Aims

#### Overview

This study has 3 aims involving assessing organizational readiness; the reach, effectiveness, adoption, implementation, and maintenance (RE-AIM) of the electronic health record–embedded individualized pain plans (E-IPPs); and barriers and facilitators of E-IPP implementation and use.

#### Aim 1

The first aim is to assess the overall effectiveness of E-IPPs in improving patient and provider outcomes associated with pain treatment in the adult emergency department setting, with the following subaims: (1) examine the effectiveness of the E-IPP on improving patients’ perceived quality of emergency department pain treatment and (2) examine the effectiveness of the E-IPP on improving providers’ self-efficacy in treating pain for patients with sickle cell disease and perceived quality of emergency department pain treatment.

#### Aim 2

The second aim is to assess the reach, adoption, implementation, and maintenance of the E-IPP components and implementation strategies at each participating site, with the following subaims: (1) assess the reach of the E-IPP, (2) assess the adoption and implementation of the E-IPP and track implementation strategies adopted by each site, and (3) assess the intent to continue using the E-IPP from a multistakeholder perspective.

#### Aim 3

The third aim is to assess organizational readiness at the beginning of implementation and barriers and facilitators to the use of E-IPPs.

## Methods

### Intervention

**Figure 1:**
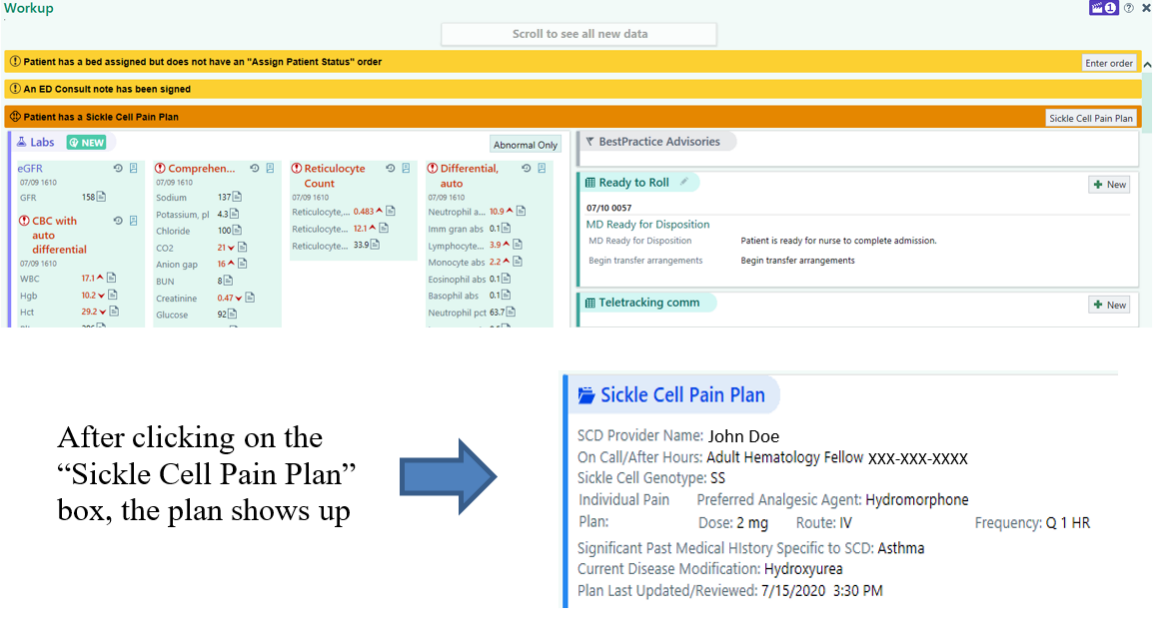
An example of an E-IPP emergency department provider interface (Washington University). E-IPP: electronic health record–embedded individualized pain plan.

**Figure 2:**
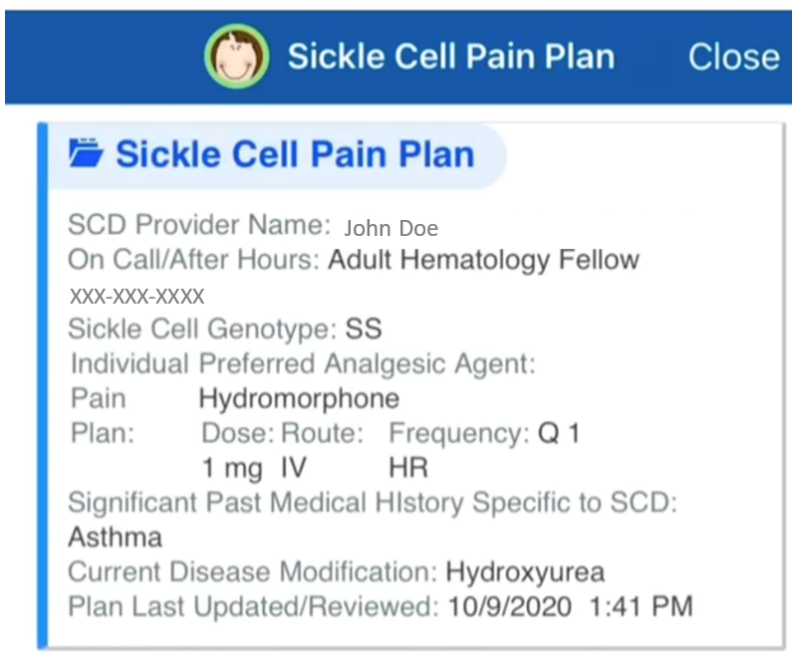
An example of an E-IPP patient portal (Washington University). E-IPP: electronic health record–embedded individualized pain plan.

The intervention for this study is E-IPPs with emergency department provider and patient access. IPPs are records developed by the sickle cell disease providers at each study site based on patients’ outpatient opioid use and analgesic agent normally required for treatment of a vaso-occlusive episode in the emergency department (Textbox 1). Each site will work with its local informatics team to make the E-IPPs available to emergency department providers via the provider EHR interface (Figure 1) and to patients via the EHR patient portal (Figure 2). The IPPs will be reviewed by the patients’ sickle cell disease providers every 6 months.

Sites will make adjustments to patients’ IPPs to ensure that all participating patients have all required content and the E-IPPs are easily accessible to emergency department providers. The E-IPPs in the patient portal will allow patients to show their IPPs as a credible source to any emergency department providers in the United States.

**E-IPP example and content. E-IPP: electronic health record–embedded individualized pain plan.**

**Required individualized pain plan contents**

- Genotype
- Individual pain plan—preferred analgesic agent, route, dose and dosing interval, last update time
- Name and contact information for the sickle cell disease provider

**Optional individualized pain plan contents**

- Allergies
- Significant past medical history specific to sickle cell disease (ie, acute chest syndrome, stroke, renal disease)
- Significant other histories relative to an emergency department visit (ie, the patient is ultrasensitive to morphine or hydromorphone)

### Study Setting

This study will take place in all 8 Sickle Cell Disease Implementation Consortium centers (Table 1). Emergency department practices vary. Prior to study implementation, 2 emergency departments had E-IPPs requiring additional content insertion or building plans for some patients, while the remainder either lacked any IPPs or only had IPPs in clinical notes or as a hard copy in a secure server not in the EHR. One of the sites had patient IPP access in the clinical notes before the study, and the rest of the sites did not have patient access to IPPs.

**Table 1:** Study site characteristics preimplementation.

Site	City	Estimated patient population	Community	Setting	Emergency department provider IPP^a^ access	Patient IPP access
Augusta University Adult Center for Blood Disorders	Augusta	358	Urban	Academic	IPPs in secured servers in the emergency department for some patients, not in the electronic health record	No
Duke University Medical Center	Durham	450	Suburban	Academic	Electronic health record–embedded IPPs, but they do not have provider contact	No
Mount Sinai Hospital	New York	175	Urban	Academic	IPPs in clinical notes	No
Methodist University Hospital	Memphis	350	Urban	Private hospital	IPPs in clinical notes	IPPs in clinical notes
Barnes-Jewish Hospital	St. Louis	300	Urban	Academic	No IPPs	No
University of California at San Francisco Benioff Children’s Hospital Oakland	Oakland	286	Urban	Academic	IPPs in clinical notes	No
University of Illinois Hospital & Health Sciences System, Sickle Cell Center	Chicago	600	Urban	Academic	IPPs in emergency department and clinical notes for select patients	No
Medical University of South Carolina Health Emergency Department	Charleston	400	Urban	Academic	Electronic health record–embedded IPPs	No

^a^IPP: individualized pain plan.

### Study Frameworks and Models

This study is guided by the RE-AIM framework, developed in 1999 to assess five dimensions (Reach, Effectiveness, Adoption, Implementation, and Maintenance) [24-26]. Each dimension of RE-AIM will be evaluated with measures at the patient, provider, or organizational and system levels where appropriate. RE-AIM was selected for this intervention because of its focus on dimensions of intervention design and implementation processes that can either facilitate or impede beneficial outcomes and can be replicated and sustained in diverse clinical settings [26,27].

**Figure 3:**
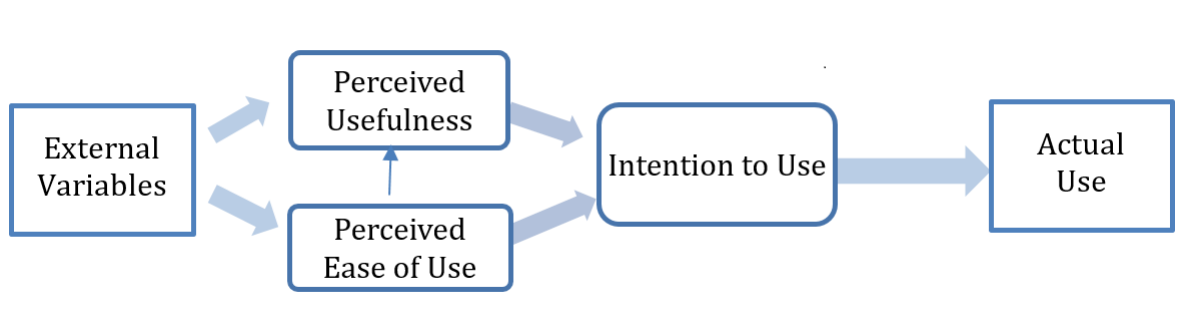
Simplified Technology Acceptance Model 2.

To complement the RE-AIM framework, this study will use a simplified version of the Technology Acceptance Model 2 (TAM2) [28] to understand E-IPP use (Figure 3). Although the TAM2 is a comprehensive model to explain technology acceptance and use, it includes additional constructs that are impractical to be measured in the emergency department setting. We have simplified the TAM2 model and will use its main constructs to help explain the intention and actual use of the E-IPPs.

The study protocol was also developed based on the Standard Protocol Items for Clinical Trials [29], a guideline for the minimum content of a clinical trial protocol, and the Standards for Reporting Implementation Studies [30], a guideline to improve the reporting of implementation studies.

### Study Design

**Figure 4:**
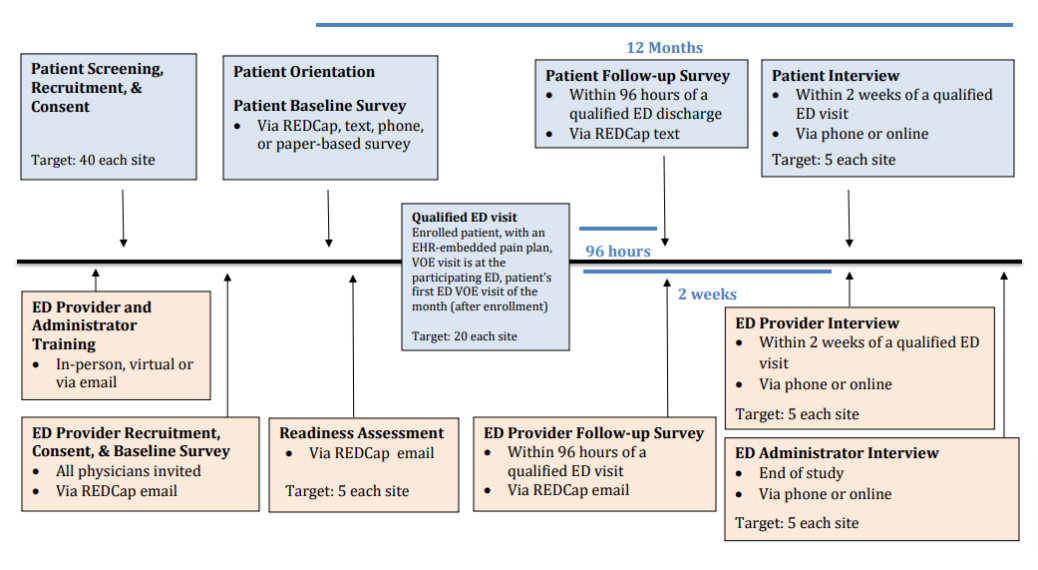
Study flow chart. ED: emergency department; EHR: electronic health record; VOE: vaso-occlusive episode.

The study uses a preimplementation and postimplementation design in which the IPPs will be embedded in patient EHRs, with both provider and patient having access to view the E-IPPs. Multimedia Appendix 1 shows the program logic model specifying the determinants, implementation strategies, mechanisms of action, and outcomes [31]. The organization of determinants is informed by the Consolidated Framework of Implementation Research, which provides a comprehensive list of constructs that would influence implementation [32], and the study’s readiness assessment, described in the following section. Figure 4 shows the study flow for each site.

Given that sickle cell disease is a rare disease and most patients with sickle cell disease do not have frequent emergency department visits per year, the preimplementation and postimplementation design without control groups is the most feasible study design. Sites will overenroll participants to ensure that enough participants will have a qualified emergency department visit during the implementation period. Additionally, it took a few months to 2 years for sites to work with their local informatics teams to make the E-IPPs available to emergency department providers and patients; therefore, a waitlist-control or stepped-wedged design is not feasible given the study timeline.

The study rollout will allow for emergency department provider training of the E-IPP, development and implementation of the E-IPPs, and evaluation postimplementation. Because of the COVID-19 pandemic, sites have adjusted to the rollout timeline. Two sites began the project in September 2020. The active implementation period is 12 months from the first day of patient enrollment, and the overall implementation phase will be 18 months.

### Implementation Strategies

Based on existing evidence, workgroup expertise, and the feasibility of implementing the E-IPP, the workgroup identified required and optional implementation strategies at the patient and provider levels to facilitate implementation and use of the E-IPP (Table 2).

**Table 2:** Implementation strategies at the patient and provider levels.

Strategy	Patient	Provider
Required	Study orientation Download and install electronic health record patient portal app Video demonstration of how to access the E-IPP^a^ Patients will be asked to show the staff how to access their pain plans via their phone (a teach-back method) Provide a wallet card with instructions to access the E-IPP and staff contact information to take home Quarterly reminder text with video demonstration	Provider training with a tracking log A 5-minute video addressing stigma and the actual prevalence of opioid addiction in sickle cell disease [33] Introduction to the study and demonstration of how to access the E-IPP
Optional	Communication script: provide a script at orientation for patients to practice how to communicate with emergency department providers about the E-IPP	Electronic health record–embedded prompts to remind emergency department triage clinicians of E-IPP Quarterly booster education sessions Disseminate and educate through podcasts, blogs, or journal clubs [34]

^a^E-IPP: electronic health record–embedded individualized pain plan.

### Participants and Eligibility Criteria

Site coordinators will keep a tracking log for patient and provider participants (Table 3).

**Table 3:** Eligibility criteria.

Entity	Criteria	
**Participating sites**		
	Inclusion	Site hematologist or sickle cell disease provider willing to write an IPP^a^ for patients meeting eligibility criteria Informatics resources available to support all aspects of the intervention Placement of the E-IPP^b^ that is accessible to both the provider and patient Ability to support text messaging to patients for survey administration Support from emergency medicine and nursing leadership to agree to follow the IPP unless there is a contraindication at the time of the emergency department vaso-occlusive episode visit
	Exclusion	None
**Patient participants**		
	Inclusion	Confirmed sickle cell disease diagnosis, defined as supported by documentation in the medical record of a positive test for 1 of the following genotypes: Hb SS, Hb SC, Hb Sβ-thalassemia, Hb SO, Hb SD, Hb SG, Hb SE, or Hb SF. If no medical record is available, the enrolling site will conduct its own laboratory test as confirmation English speaking Age 18-45 years (National Heart, Lung, and Blood Institute grant requirement) Access to either an Android or iOS cellular or mobile smartphone, with access to text messaging and internet At least 1 vaso-occlusive episode visit to the participating site’s emergency department within the past 90 days prior to enrollment At least 1 visit at the study site sickle cell disease clinic within the past 12 months Willing and cognitively able to give informed consent
	Exclusion	Site hematologist or sickle cell disease provider states patient should not have a protocol or should not be administered opioids
**Provider participants**		
	Inclusion	All emergency physicians, nurses, physician assistants, and nurse practitioners who work in the study emergency department will have access to the E-IPPs as routine practice and physicians are asked to complete baseline surveys at enrollment for research purposes
	Exclusion	None

^a^IPP: individualized pain plan.

^a^E-IPP: electronic health record–embedded individualized pain plan.

### Recruitment

#### Patient Recruitment and Procedures

Potential participants will be approached in person, by phone, or via electronic media about enrolling in the study. All patients enrolled in the Sickle Cell Disease Implementation Consortium registry and screened as eligible will be contacted by research staff for participation in the emergency department project. Additional participants meeting criteria at participating Sickle Cell Disease Implementation Consortium sites not previously enrolled in the registry are also eligible and may be recruited in the clinic, during hospitalizations, or at community events by research staff. Patient participants will provide informed consent before study participation.

#### Provider Recruitment

All emergency department providers will be able to access the E-IPPs and will receive training on the protocol at staff meetings, resident conferences, or through emails. All emergency department providers will be invited to complete a baseline survey at enrollment with an information sheet introducing the study. If an enrolled patient participant has a qualified emergency department visit and the corresponding emergency department provider has not received training or completed the baseline survey, the study team will send a provider follow-up survey to the provider at enrollment with an information sheet introducing the study.

A qualified emergency department visit is defined as (1) a visit for an enrolled patient to a participating emergency department, the reason for the visit is a vaso-occlusive episode, and the patient has an E-IPP at the time of the emergency department visit; and (2) the patient’s first emergency department vaso-occlusive episode visit of the month, after enrollment, within the 12 month study period for that patient.

### Data Collection

The data collected in this study will consist of a readiness assessment; EHR data retrieval from patient records; patient baseline and follow-up surveys; provider baseline and follow-up surveys; and patient, provider, and emergency department administrator interviews. All measures (other than the readiness assessment) are matched with the RE-AIM framework (Multimedia Appendix 2).

### Readiness Assessment

The participating centers will administer a Readiness Diagnostic Scale (RDS) developed by members of the Consortium’s Implementation Research Committee at the beginning of the implementation period [35]. The RDS is a quantitative assessment to capture 3 interrelated dimensions of organizational readiness: general capacities (34 items), assessing participating emergency department’s existing emergency department practice and how it functions overall; intervention-specific capacities (13 items), assessing participating emergency department capacity to use the E-IPP; and motivation (17 items), assessing how well the emergency department facilitates physicians’ willingness to use the E-IPP.

Each site is required to have at least 5 total completed RDSs from a study team researcher, emergency department administrator, emergency department physician, and emergency department nurse to capture different system-level roles in clinical care.

### EHR Data Retrieval

Each participating site will perform EHR data retrieval for enrolled patients’ past 12 months ED visits and qualified ED visits to collect: hospital admission rate; 7- and 30-day emergency department revisit rate; 7- and 30-day hospital readmission rate; time to first dose of analgesic agent; and administered analgesic agent, dose, and route. At the end of the project, the site will also retrieve the following from the EHR: number of E-IPPs written and time of writing or updating, number of new E-IPPs available in the EHR and time of becoming available, and number of new patients at each site who are being offered access to their pain plan in the EHR after study enrollment targets are met. Data retrieval will follow a manual of procedures created by the workgroup to reduce bias [36,37].

### Patient Baseline and Follow-up Survey

Each participating site will administer a brief patient baseline survey at the time of patient enrollment that will assess patient demographic data, patient-perceived quality of emergency department pain treatment for the last emergency department visit (within 90 days of enrollment), and how well the patient knows how to use the patient portal. Within 96 hours of a qualified emergency department visit, the research team will send a follow-up survey by text message to the patient that will assess patient’s perceived quality of emergency department pain treatment for this visit, how well they know how to use the patient portal, perceived ease of use of the E-IPP, patient and emergency department provider use of E-IPP during the last visit, satisfaction with the E-IPP, and intent to use the E-IPP for next emergency department vaso-occlusive episode visit. Patients will receive up to 3 follow-up surveys during the study period.

### Patient Interview

After a center has had 5 patients with qualified emergency department visits, its team will begin to invite patients for interviews within 2 weeks of a qualified emergency department visit. The patient interview will assess the patient’s experiences using (or not using) the E-IPP, what was helpful and challenging about using the E-IPP, if pain treatment has changed (or not changed) because of the E-IPP, patient satisfaction with the E-IPP, proposed recommendations to improve the E-IPP, intent to use the E-IPP in the future, and additional questions on implementation strategies such as the wallet card. The research team will use purposeful sampling for qualitative data collection [38], based on matched patient and provider survey responses and implementation outcomes of the IPPs.

### Provider Baseline and Follow-up Survey

Each participating site will administer a brief provider baseline survey at the time of provider enrollment that will assess provider-perceived quality of emergency department pain treatment and provider’s self-efficacy to manage acute pain episodes for patients with sickle cell disease. Within 96 hours of a qualified emergency department visit, the research team will send a follow-up survey via email to the enrolled provider who ordered the first analgesic. The provider follow-up survey will assess use of E-IPP, ease of E-IPP use, E-IPP adherence, perceived quality of emergency department pain treatment, satisfaction with the E-IPP, and intent to use the E-IPP in the future.

### Provider Interview

After a center has had 5 patients with qualified emergency department visits, the team will begin to invite providers who ordered the first analgesic for interviews within 2 weeks of a qualified emergency department visit. The provider interview will assess provider’s experiences using (or not using) the E-IPPs, what was helpful and challenging about using the E-IPPs, proposed recommendations to improve the E-IPPs, intent to use the E-IPP in the future, and additional questions on implementation strategies. The research team will use purposeful sampling for qualitative data collection [38], based on matched patient and provider survey responses and implementation outcomes of the E-IPPs.

### Emergency Department Administrator Interview

At the end of the implementation period, each site team will invite emergency department administrators to a postimplementation interview that will assess barriers and facilitators to intervention implementation, emergency department administrator’s experiences implementing and using the E-IPP, and emergency department administrator’s intent to continue using the E-IPP in the future.

### Tracking and Reporting Implementation Strategies

A parallel study has been funded to capture the planned and actual implementation strategies employed by each site [39]. Semistructured interviews with 3 stakeholders from each site before participant recruitment and at the end of the intervention, as well as quantitative surveys at the midpoint of project implementation, will capture the barriers and facilitators of the strategies planned and whether there have been any adaptations made. Questions have been informed by the implementation and maintenance domains of the RE-AIM framework.

### Statistical Analysis Power and Sample Size

The study plans to have an analytical sample size of 160 patient participants, 20 for each site, for its primary outcome analysis. Each site is expected to enroll approximately 40 participants to reach the analytical sample size.

Sample size was calculated for the primary outcome, patient satisfaction with emergency department pain treatment through their perceived quality of emergency department pain treatment. For the power calculations, we used results from the Sickle Cell Disease Implementation Consortium needs assessment [17, 23], which used similar measurements to assess patient satisfaction with emergency department pain treatment. We assumed that the correlations between responses would not change and conducted a simulation to determine statistical power to detect a treatment difference of half a standard deviation. The sample estimation model has several assumptions: the intervention effect will vary across sites with the standard deviation for the random intervention effect set at 0.447; within-site correlation (commonly referred to as intercluster correlation) of baseline measurements, calculated as the ratio of site-level variance over the total variance, is set at 0.10; and within-participant correlation, calculated as the ratio of individual-level variance over total individual variance, is set at 0.50, reflecting the expectation that the preintervention composite score is predictive of the postintervention score.

Based on the Sickle Cell Disease Implementation Consortium registry data, we estimated that at least 50% of the study patient participants will have at least 1 qualified emergency department visit during the study period and that approximately 20% of the participants will be lost to follow-up and provide no posttest data. The total number of patients a site will recruit is approximately 40. The results indicate that the study will have >90% power to reject the null hypothesis when the intervention results in an average improvement in patient-perceived quality of emergency department care of 0.5 standard deviations under the assumed conditions.

### Data Analysis

#### Primary Outcome

The primary outcome is patient satisfaction with emergency department pain treatment through their perceived quality of emergency department pain treatment (Multimedia Appendix 2). This is measured with a composite of 3 questions developed based on the Adult Sickle Cell Quality of Life Measure Quality of Care measure [6]. The impact of the intervention on the primary outcome will be estimated using a linear mixed model. A 3-level model with random effects for site, participants nested within site, and site-to-site variation in the treatment effect and a fixed effect for treatment response will be specified to assess the impact of the intervention. The primary analysis will be based on the preintervention composite score and the score at 1 emergency department visit per participant, using the first visit for those with more than one. The model can accommodate multiple visits per participant by adding a covariance matrix to account for repeated observations.

#### Other Outcomes

The secondary outcomes of hospital admission rate (a count outcome, as multiple hospital admissions are possible), 7-day emergency department vaso-occlusive episode revisit (a dichotomous outcome), and time to first dose, and other provider-level outcomes will be analyzed with generalized linear mixed models, where the outcome will be specified as a Poisson or binomial variable with a logit function as needed by the data type of outcome. The generalized linear mixed models would follow the same format as the linear mixed model with fixed effects for time, intervention, and covariates and random effects for participants within sites. The secondary outcome of satisfaction with IPP, measured with a single Likert scale question for both patient and provider, will be analyzed as a continuous outcome with a linear mixed model as detailed above for the primary outcome. In all cases, the analysis will estimate an intervention effect that will compare the secondary outcome at baseline to after the intervention is delivered. Power calculations indicate that the study sample will be too small to detect statistically significant differences in these noncontiguous variables, but estimates will be provided to inform future research.

The analysis of the quantitative readiness assessment result and the reach, adoption, implementation, and maintenance outcomes will be descriptive. As a preliminary attempt, at the end of the intervention, the results of the readiness assessment will inform our interpretation of the implementation and effectiveness outcomes. Sites with a lower level of readiness may face challenges during implementation, which will affect their implementation outcome and intervention effectiveness.

For qualitative interviews, the workgroup members will develop a codebook informed by the RE-AIM framework for data analysis. Using a deductive approach, the codebook will include an initial list of codes to be used in the analysis and definitions or operationalized examples for each code. Analysts will revise the codebook as necessary to hone definitions to increase consistency in coding across the research teams. Data will be compiled into different stakeholder groups by themes and analyzed across the study sites.

## Results

At the time of submission, this study was approved by the institutional review board of Duke University (IRB Pro 000073506), Icahn School of Medicine at Mount Sinai (IRB 20-04302), Medical University of South Carolina (IRB Pro 00097830), Washington University School of Medicine (IRB 202006209), University of California San Francisco (IRB 20-30981), University of Illinois Chicago (IRB 2016-02998). The full protocol and study manual of procedures are available from the corresponding author upon request. The study is registered (NCT04584528) and enrolling participants. Sites that did not have IPPs for all patients are working with the sickle cell disease providers to build the plans with patients during clinic visits. Because of the COVID-19 outbreak, sites have changed the planned recruitment timeline and will continue enrolling until the target is reached.

## Discussion

With the rapid expansion of the use of EHR in the past decade, building an EHR-embedded treatment plan for different patient populations has gained increased interest, especially in the emergency department, where decision making is time sensitive. Previous research has explored various decision support tools, including treatment plans, to facilitate emergency department provider decision making [11,40-42]. The E-IPP intervention is a direct result of a large-scale needs assessment effort and is designed to meet the needs of participating sites and patient populations. Guided by the RE-AIM theoretical framework and a widely used TAM2 model explaining technology uptake, the protocol proposes to test the effectiveness of the E-IPP while also describing how the intervention is implemented and assessing implementation outcomes in detail.

Previous research on individualized treatment plans for patients with sickle cell disease has largely been conducted in pediatric settings [9-11]. These studies demonstrated that providers perceived that digitization of the treatment plans could help improve the efficiency and quality of pain management [9-11]. So far, only 1 randomized controlled trial [12] has tested the effectiveness of an individualized treatment plan compared to a weight-based plan for adult patients with sickle cell disease and showed significant efficacy, but the results were not generalizable, as it was conducted in only 2 emergency departments with 52 patients. While previous studies have focused on assessing patient outcomes, this study will assess multilevel outcomes, including patient experience and satisfaction. With a comprehensive understanding of the uptake and implementation of the E-IPPs, the intervention will be more likely to successfully be disseminated to other emergency departments on a large scale.

The most novel component of this protocol is making the E-IPP available to the patient. Many sickle cell centers already make IPPs available to the emergency department provider, but few have been able to make these accessible to the patient. Among 8 Sickle Cell Disease Implementation Consortium sites, only 1 site had patient IPP access through the clinical notes. With this project, patients will have easy E-IPP access and will be able to present their IPPs to the emergency department provider directly via the patient portal app on their cellular device. In the past decade, smartphone ownership has increased significantly in the United States, even among low- and moderate-income communities [43]. Over 70% of individuals with an annual income of less than $30,000 reported smartphone ownership in 2019, and the rates of smartphone ownership among patients with sickle cell disease are similar [43-45]. Previous studies [46-48] have identified barriers to patients accessing and using an EHR, and participants with limited health literacy may need additional time to navigate the EHR patient portal. Previous literature has demonstrated substantial disparities in portal use, indicating that vulnerable populations, such as racial and ethnic minorities and individuals with low socioeconomic status, are less likely to use patient portals, which is relevant for sickle cell disease, as the majority of individuals living with sickle cell disease are African American [49,50]. This study will accommodate many barriers by providing a one-on-one session to help patients install the patient portal for E-IPP access and by providing a wallet card with instructions and videos of how to access their E-IPPs. As previous exploratory research on disease-specific patient portals in patients with sickle cell disease has achieved high acceptability, the E-IPPs, within a few clicks from the main menu, are likely to be viewed favorably by study participants [50]. The study will be able to explore whether patient access to E-IPPs will help with their treatment experiences in the emergency departments.

For the protocol, a significant challenge is to balance the desire for robust study design with practical needs in the emergency departments, which is common for implementation research studies [51]. Acknowledging that a preimplementation and postimplementation study design without control groups has limitations, it is the only feasible solution given that sickle cell disease is a rare disease and study sites must overenroll participants to collect data on enough emergency department visits to generate meaningful results. All emergency department sites want to adopt E-IPP implementation to improve practice as soon as possible. The protocol uses comprehensive mixed methods data collection [52] to compensate for the study design to achieve the 3 study aims. Some of the activities will be exploratory in nature and provide preliminary data to inform future work expanding the E-IPP on a larger scale and beyond academic settings.

Another significant challenge is that the Sickle Cell Disease Implementation Consortium consists of 8 academic sites, and local practices vary. Because the EHR systems may be structured in different ways, sites have spent an extensive amount of time working with their local informatics teams to make the E-IPPs available in both patient and provider channels. The protocol has adopted several measures to capture and address this diversity, including surveying sites about their existing practices, incorporating the readiness assessment component to help sites understand capacity and motivations that may be unique to their site, and a separate protocol to solely focus on implementation strategies and adaptations at the site level.

In summary, this study proposes a framework-informed, structured approach to implement a guideline recommendation in routine emergency department practice. It is the first multisite study investigating the effectiveness of E-IPPs in adult emergency departments at both the provider and patient levels. The results of the study will inform the implementation of IPPs with emergency department provider and patient access at a larger scale.

## Appendix

Multimedia Appendix 1 Program logic model of the E-IPPs. ED: emergency department; EHR: electronic health record; E-IPP: electronic health record–embedded individualized pain plan; IPP: individualized pain plan; SCD: sickle cell disease.

Multimedia Appendix 2 Outcomes and the RE-AIM evaluation framework.

Multimedia Appendix 3 List of Sickle Cell Disease Implementation Consortium investigators.

Multimedia Appendix 4 NHLBI Peer-review Report, with comments and responses.

## Abbreviations

EHR: electronic health record
E-IPP: electronic health record–embedded individualized pain plan
IPP: individualized pain plan
RDS: Readiness Diagnostic Scale
RE-AIM: Reach, Efficacy, Adoption, Implementation, and Maintenance Model
TAM2: Technology Acceptance Model 2
