# Supplementary figures and images for: Electronic Health Record–Embedded Individualized Pain Plans for Emergency Department Treatment of Vaso-occlusive Episodes in Adults With Sickle Cell Disease: Protocol for a Preimplementation and Postimplementation Study
Source: JMIR Res Protoc. 2021 Apr 16;10(4):e24818. doi: 10.2196/24818 (PMC8087964; doi:10.2196/24818)

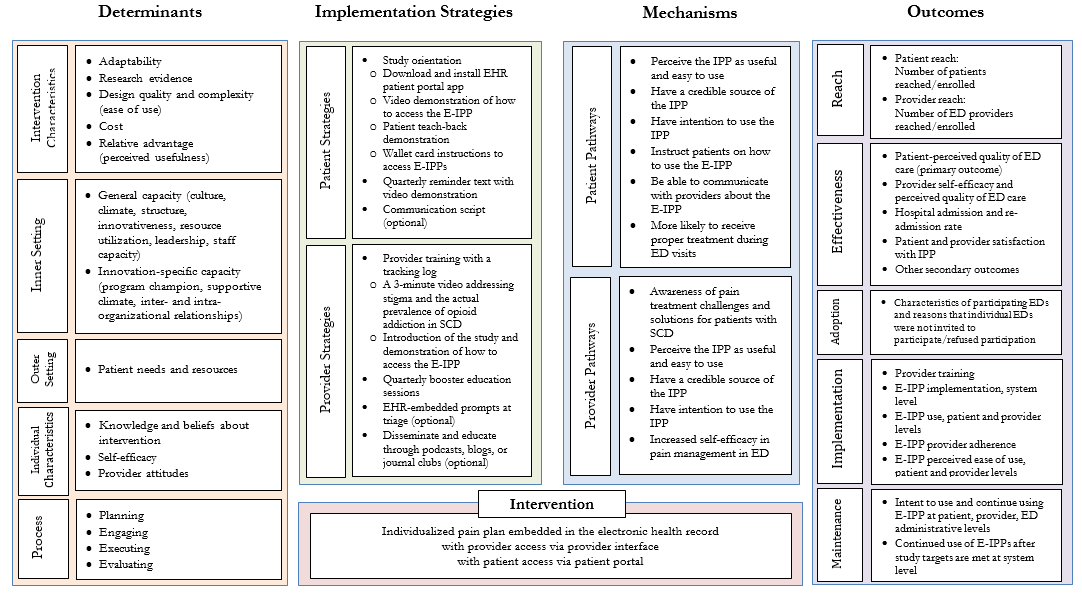

Supplement: Multimedia Appendix 1 [file resprot_v10i4e24818_app1.png]
