# Supplementary material for: Electronic Health Record–Embedded Individualized Pain Plans for Emergency Department Treatment of Vaso-occlusive Episodes in Adults With Sickle Cell Disease: Protocol for a Preimplementation and Postimplementation Study
Source: JMIR Res Protoc. 2021 Apr 16;10(4):e24818. doi: 10.2196/24818 (PMC8087964; doi:10.2196/24818)
Supplement: Multimedia Appendix 2 [file resprot_v10i4e24818_app2.docx]

| RE-AIM  Domain and Measurement | Level of Measurement | Measure | Baseline/Enrollment | Post–qualified ED visit | | 12 Months of Prospective Enrollment |
| --- | --- | --- | --- | --- | --- | --- |
|  |  |  |  | Within 96 hours | Within 2 weeks |  |
| **Reach** | | | | | | |
| **Patient reach** | Patient | Proportion of patients approached (denominator) and actually enrolled (numerator) | Research team tracking | | | |
| **Provider reach** | Provider | Proportion of ED providers approached (denominator) and actually enrolled (numerator) | Research team tracking | | | |
| **Effectiveness** | | | | | | |
| **Patient-perceived quality of ED pain treatment** | Patient | **[PRIMARY OUTCOME]**  Composite measure of three questions from the needs assessment (5-point scale)   1. Were you satisfied with the care you received? 2. How much were the emergency room doctors and nurses able to help your pain? 3. How much did the emergency room doctors and nurses believe you had very bad sickle cell pain? | Patient  baseline  survey | Patient follow-up survey | Patient interview |  |
| **Provider self-efficacy & perceived quality of ED pain treatment** | Provider | 1. Self-efficacy  2. Perceived quality of ED pain treatment | Provider  baseline  survey | Provider  follow-up  survey | Provider  interview |  |
| **Hospital admission rate** | System/site | All enrolled patients, VOE visits;  Hospital admission rate | EHR retrieval,  past 12 months |  |  | EHR retrieval,  past 12 months |
| **Other secondary patient outcomes** | System | All enrolled patients, VOE visits   1. 7- and 30-day ED revisit rate 2. 7- and 30-day hospital re-admission rate 3. Time to first dose | EHR retrieval,  past 12 months |  |  | EHR retrieval,  past 12 months |
| **Satisfaction with IPP** | Patient | On a scale of 1–7, how helpful was the pain plan in helping you get the pain treatment you needed? (1 = not at all, 7 = excellent) |  | Patient follow-up survey | Patient  interview |  |
|  | Provider | On a scale of 1–7, how helpful was the IPP in providing care to the patient with SCD? |  | Provider follow-up survey | Provider  interview |  |
| **Adoption** | | | | | | |
| **IPP adoption** | System/site | Characteristics of participating EDs and reasons that individual EDs were not invited to participate or refused participation | Research team tracking |  |  |  |
| **Implementation** | | | | | | |
| **Provider training** | Provider | Proportion and representativeness of ED providers who completed a training session (numerator) among all ED providers at each site (denominator).  A tracking log of provider training containing who, when, and where the providers completed the training with provider unique identifying ID | Research team tracking |  |  |  |
| **IPP implementation** | System | 1. Number of IPPs written and when  2. Number of new IPPs available in the EHR and when |  |  |  | EHR review;  Research team tracking |
| **IPP implementation (parallel study)** | System | Number of required and optional program elements implemented as planned in each site, reasons they were or were not implemented, and changes made during implementation | A supplement study—interview site members  one month into the implementation & at end of the implementation | | | |
| **IPP use** | Patient | IPP use |  | Patient follow-up survey | Patient interview |  |
|  | Provider | IPP use |  | Provider follow-up survey | Provider interview |  |
| **IPP adherence** | System/ED | 1. Number of ED VOE visits when IPP is accessed/total participating sites’ ED VOE visits when patients have IPP in EHR  2. Adherence to correct drug (yes/no); dose (within X morphine sulfate equivalents); route |  | Provider follow-up survey | Provider interview | EHR review |
| **Perceived ease of use of IPP** | Patient | TAM2 measurement  Likert 1–7 |  | Patient Follow-up Survey | Patient interview |  |
|  | Provider | TAM2 measurement  Likert 1–7 | Provider  baseline  survey | Provider follow-up survey | Provider interview |  |
| **Maintenance** | | | | | | |
| **Intent to use/**  **continue to use IPP** | Patient | Intent to use   - TAM2 measurement - Likert 1–7   Intent to continue using   - TAM2 measurement with revisions - Likert 1–7 - Additional qualitative questions |  | Patient follow-up survey | Patient interview |  |
|  | Provider | Intent to use   - TAM2 measurement - Likert 1–7   Intent to continue using   - TAM2 measurement with revisions Likert 1–7 - Additional qualitative questions | Provider  baseline  survey | Provider follow-up survey | Provider interview |  |
|  | ED administrators (medical and nursing directors) | Intent to continue using IPP qualitative questions  (i.e. do the EDs want to continue using the program, and how would they want to adapt the program if they were to try to scale it up?) |  |  |  | ED administrator interviews |
| **Continued use of IPP** | System | Number of new patients at each site who are being offered access to their pain plan in the EHR after study targets are met |  |  |  | EHR review |
