# Supplementary material for: Electronic Health Record–Embedded Individualized Pain Plans for Emergency Department Treatment of Vaso-occlusive Episodes in Adults With Sickle Cell Disease: Protocol for a Preimplementation and Postimplementation Study
Source: JMIR Res Protoc. 2021 Apr 16;10(4):e24818. doi: 10.2196/24818 (PMC8087964; doi:10.2196/24818)
Supplement: Multimedia Appendix 3 [file resprot_v10i4e24818_app3.docx]

**St. Jude Children’s Research Hospital, Memphis, TN**

Jane Hankins, MD, MS

Jason Hodges, PhD, MA

Yvonne Carroll, RN, JD

Lisa Klesges, PhD, MS

Hamda Khan, MA

Matthew Smeltzer, PhD, MS

Chinonyelum Nwosu, MPH

James Gurney, PhD

Jerlym Porter, PhD, MPH

Nicole Alberts, PhD

Jeffrey Frey, BS, MS, MBA, PhD

Reginald French

Michael Jackson

Michael DeBaun, MD, MPH

Ramin Homayouni, PhD

Tamanna Shamrin, PhD, MS

Guolian Kang, PhD

Jeremie Estepp, MD

Winfred Wang, MD

Artangela Henry, DNP

Curtis Owens, MD

Margaret Debon, PhD

Ray Osarogiagbon, MD

Nidhi Bhatt, MD

Justin Flowers

**University of California, San Francisco, CA**

Marsha Treadwell, PhD

Elliott Vichinsky, MD

Ted Wun, MD

Michael Potter, MD

Danielle Hessler, PhD

Ward Hagar, MD

Anne Marsh, MD

Lynne Neumayr, MD

**University of South Carolina, Charleston, SC**

Cathy Melvin, PhD

Julie Kanter, MD

Shannon Phillips, PhD, RN

Robert Adams, MD

Martina Mueller, PhD

**DUKE University, Durham, NC**

Nirmish Shah, MD

Paula Tanabe, PhD, MSN

Hayden Bosworth, PhD

George Jackson, PhD

Fred Johnson, MBA

Rachel Richesson, PhD

Janet Prvu-Bettger, ScD

Lauren Siewny, MD

**Washington University, St. Louis, MO**

Allison King, MD, PhD

Lingzi Luo, MPH, MSW

Ana Baumann, PhD

Christopher Carpenter, MD

CeCe Calhoun, PhD

Aimee James, PhD, MPH

Regina A. Abel, PhD

**AUGUSTA University, Augusta, GA**

Richard Lottenberg, MD

Abdullah Kutlar, MD

Robert Gibson, PhD

Angie Snyder, PhD

Maria Fernandez, PhD

Betty Pace, MD

E. Leila Jerome Clay, MD

Raymona Lawrence, DrPH

**Ichan School of Medicine at Mount Sinai, New York, NY**

Lynne D. Richardson, MD

Jeffrey Glassberg M.D. M.A.

Jena Simon, MS, APRN-BC

Nicholas G. Genes, MD, PhD

George T. Loo, DrPH

Jason S. Shapiro, MD, MA

Kimberly Souffront PhD, FNP-BC, RN

Cindy Clesca, MA

Elizabeth Linton, MPH

Gery Ryan PhD, MA

**RTI INTERNATIONAL**

Barbara L Kroner, PhD

Lucia Rojas-Smith, DrPH

Tabitha Hendershot, BA

Lisa DiMartino, PhD, MPH

Lisa Cox, MS

Sara Jacobs, PhD

Whitney Battestilli, BA

Donald Brambilla, PhD

Liliana Preiss, MS

Norma Pugh, MS

**NHLBI, Bethesda, MA**

Sharon M Smith, PhD

Harvey Luksenberg, MD

Marlene Peters-Lawrence, BSN, RN

Cheryl Boyce, PhD

Ellen Werner, PhD

**LURIE CHILDRENS, Chicago, IL**

Alexis Thompson, MD

**University of Chicago, Chicago, IL**

Kim Erwin, MDes

Andrea Lamont, PhD

Molly Martin, MD, MAPP

Sarah Norell, MDes, MFA

Ananta Pandit, MD

Kay Saving, MD

Robin Shannon, DNP, RN

Robert Winn, MD

Leslie Zun, MD

Taif Hassan, MD

Patricia Lasley, MPH

Kristin Monnard, MPH

Judith Nocek, PhD

Pamela Roesch, MPH
