# Supplementary material for: Electronic Health Record–Embedded Individualized Pain Plans for Emergency Department Treatment of Vaso-occlusive Episodes in Adults With Sickle Cell Disease: Protocol for a Preimplementation and Postimplementation Study
Source: JMIR Res Protoc. 2021 Apr 16;10(4):e24818. doi: 10.2196/24818 (PMC8087964; doi:10.2196/24818)
Supplement: Multimedia Appendix 4 [file resprot_v10i4e24818_app4.pdf]

NHLBI / SCD-SCDID OSMB Protocol Review Form, Reviewer 1-3 compiled, Nov 26.
**Sickle Cell Disease Implementation Consortium**<br>**Implementing an Individualized Pain Plan (IPP) with Patient and Provider Electronic Health Record Access, for Adult Emergency Department Treatment of Vaso-occlusive Episodes in Sickle Cell Disease: A Pre-post Study Design**

Instructions: This form is designed to guide protocol reviews and to collect comments, issues of concern, requests for additional information or clarification, and to record an initial approval recommendation. Please forward the completed form to [redacted] y Tuesday, November 19, 2019

Study Objectives and Feasibility	Acceptable	Needs Discussion	Not Reviewed
Adequacy of previous research and/or clinical studies of proposed treatment or therapy	3		
Scientific merit of the study objectives; primary and secondary endpoints	2	1	
Interventions: treatment or therapeutic regimen	3		
Data to be collected: laboratory tests, clinical exams, outcome(s) of interest	2	1	
Feasibility: including recruitment strategies, target recruitment goals, and timetable for study completion	3		

COMMENTS:

Study Design and Statistical Approach	Acceptable	Needs Discussion	Not Reviewed
Study hypothesis(es)	3		
Experimental design: sample size, use of controls or standard treatment or therapy, single/multicenter design	1	2	
Statistical approach, power and data analysis plan	3		

COMMENTS:

Study Operations	Acceptable	Needs Discussion	Not Reviewed
Plans for study operations: organization of clinical centers, governing committees, data coordinating center	3		
Plans for training clinical center personnel: good clinical practice, research goals, protocol details and implementation, and study operations	2	1	
Plans for data acquisition: data management, quality assurance, and ultimate transfer of data to NHLBI	2	1	

COMMENTS:

1. **Will fidelity be assessed?**
   Yes. We will assess implementation fidelity in the following ways:

1) *Implementation strategies: we will track implementation strategies planned and actually used by each site. We will also track implementation strategies and their specifications (e.g. dose, who performed the strategy, where it was done) either through tools (e.g. provider training log) or through the parallel supplement study's interviews. (Table 4, Implementation, Provider training, & IPP implementation parallel study (Baumann, Belle, James, & King, 2018)).*
2) *Implementation outcomes: we will assess to what extent the providers use the IPP, including whether they used it or not in the ED visit via survey and interview, and we will retrieve information from the EMR and compare the drug and dose ordered during an ED visit to the drug and dose given during an ED visit. (Table 4, Implementation, IPP Use & Adherence).*

2. **Will you keep track of demographics of participants who decide not to participate; how about dissemination to the clinics and patients?**
   We will keep a screening log at each site for individuals we reach out to consent, including name, date and method of contact, and date of consent or refusal. We can also note why they refuse to participate in the screening log. However, IRB does not allow obtaining or retrieving demographics of those who refuse to participate in the study, so no other demographics of individuals who decide not to participate will be documented.

   In terms of dissemination to the clinics and patients, all sites have hematologists on the research team or in collaboration. The protocol will be shared with hematologists at each participating center. Local ED physicians will be aware of the protocol, and local community based organizations will be aware of the opportunity to participate. Recruitment and consent will take place in the primary hematology clinic. The SCD clinic hematologist will be writing the IPP and the IPP will be reviewed with the patient at enrollment.

3. **Any way rural settings can be included?**
   None of the 8 centers in the SCDIC are considered to be a rural setting. The intervention requires local team to work heavily with the site informatics team and ED providers to work out how IPP will made available in the EHR and this process is very site specific. At this time we are unable to include additional sites. However, several of the sites serve patients who live in rural settings. Their ED care will be in the suburban or urban medical center.

4. **How about reach at the staff and clinic levels?**
   This is an ED focused implementation intervention, so we are not measuring the reach of the sickle cell clinic staff. The clinic SCD provider will be writing the IPP, representing 100% reach of the participating sickle cell clinics in the eight centers.

5. **In pilot testing, make sure 5 patient represent demographics of the group you are targeting. Should pilot testing include more than 3 ED providers (include staff too: front desk, nurse). For the pilot testing phase—make sure wallet instructions for patients is health literacy friendly.**
   We will include an ED nurse in the pilot phase. We will not include front desk staff as they will not be directly involved in the intervention.

Human Subjects Protection	Acceptable	Needs Discussion	Not Reviewed
Model informed consent document	**1**	**2**	
Data and safety monitoring plan: including adverse event notification, medical monitor, and stopping guidelines	**3**		
Subject population: including appropriate representation of minorities, women, and children	**3**		

Discussion of Equipoise	**3**		

Approval Recommendation	Approve	Approve with Changes*	Do Not Approve	Postpone Approval Recommendation Until After Discussion
Protocol	**1**			**3**
Model Consent Document	**1**	**1**		**1**
Other materials	**2**			

***Please describe the changes deemed necessary for approval:**

1. **Major concern is the primary outcome which is the change in the composite score of patient perception of quality of pain management in the ED setting. The investigators are going to use as a baseline patient recall of their experience in the ED in the 12 months prior to enrollment and compare that to a survey done within 72 hours of a study visit during the study period. I have two concerns with this: one is that I am not sure they are measuring the same thing as for the baseline patients are being asked to recall their experience. For some patients this might be recalling one episode of ED care and for others it could be recall of many ED visits. The investigators are then going to compare this recall assessment with a survey done at the time of a visit. The investigators state that a change of 0.5 SD would be clinically significant but it is unclear how they came to that conclusion.**
   The study team discussed the first concern at length. We chose to phrase the question of recall of 12 months, vs. the last visit, because many patients will typically remember only the "worst" visit, which in some cases, could have been 10 years prior. We have no guarantee that patients will interpret as we describe, but a script of how to ask this question when enrolling patients will be developed. This will be critical. We do think it is valid to compare a baseline, overall quality of pain management variable to measure changes with future visits. Essentially, patients are being asked to report, their perception of pain care over the past year, with a most recent visit, within 72 hours, for patients that have an ED visit after enrollment.

   The study team, including the statistician, discussed the 0.5 SD change at length. Given the distribution of the scale, we agreed that a 0.5 change in SD would represent a significant change in the perception of quality of care.

2. **It is also unclear that this tool has been validated as a measure of the quality of pain management in the ED.**
   The team has discussed this at length and due to the limitations of conducting this difficult protocol in an ED setting, we believe that obtaining patient reported perception of the quality of ED pain care within 72 hours of an ED visit is a more than acceptable proxy measure for pain relief. We are happy to discuss further.

   As stated in the protocol "Quality of ED pain care in this study is the primary outcome because quality of pain care is reflective of how well the pain was managed and IPP's are the gold standard for VOE management. The most precise outcome for this study would be the direct measure of pain scores from the patient at arrival and discharge. It is not possible to rely on electronic health record documentation of pain scores because typically >50% of pain scores are missing, especially at discharge. To obtain pain scores prospectively from the patient would require the presence of research staff in the ED 24/7. Typically, there

are between 0-3 ED visits/day for VOE; thus collection of pain scores is not within the capacity of this project."

The questions are validated and were the same questions used to conduct the needs assessment for the SCDIC initial stage fo the project. These questions are from the Adult Sickle Cell Quality of Life Measure (ASCQ-Me), Quality of Care domain. This instrument is a QoL tool developed for SCD and un-gone rigorous psychometric testing. Our group has discussed this at length and selected these 3 questions as the most validated questions that exist for measuring pain management in the ED.

3. **There are several interventions including a 2 minute video on stigma. How will the investigators know whether it was the IPP or the other interventions that effected the quality of pain management? Especially since only some of the sites will be doing booster education.**
We are implementing one intervention - the individualized pain plan embedded in the EHR. There will be many actions or activities taken, which are implementation strategies, defined as 'activities that each site will do to support the implementation of interventions' in our protocol page 14. Having the education materials, including the 2 minute video on stigma, is one of our core implementation strategy to support the intervention. We acknowledge that some components of the implementation strategies, such as the 2 minute video on stigma, might be considered an intervention to affect quality of pain treatment. The same goes with other strategies, such as engaging providers to participate in the study might increase their awareness, improve their attitude, and affect quality of pain treatment. This is an implementation research study, it is expected that sites will have adaptations for implementation strategies based on their needs and resources, it is impossible to have all sites performing all the same strategies, and we will track strategies and their specifications of each site. The intervention should not be viewed without context and without these implementation strategies. In addition to tracking which sites use the 2 minute video, each site will track any other strategies performed during the data collection period that may affect the primary outcome. Each site will conduct interviews or focus groups at the end of the data collection period with key stakeholders from each site. Stakeholders will include ED administrators (medical and nursing leadership) and discuss any additional strategies that may have been implemented, in addition to the study interventions, which may have affected the study outcomes.

4. **Will all data on all visits to the ED be included – what if patient is seen for something more than an uncomplicated VOE?**
Please refer to the definition of 'a qualified ED visit' on protocol page 16, we will only include data on ED visits for VOE reasons.

5. **It appears that some patients already have pain plans. One has to assume that at least some of those pain plans have been used in the past so when those patients reflect on their prior 12 months in the ED it might be influenced by the fact that they have an existing pain plan- how will the investigators deal with this issue when looking at outcomes?**
Many sites do not already have pain plans, so new pain plans will be developed for several sites. The dataset will include a variable, "existing IPP", in the dataset. We will conduct an analysis to measure the effect of pre-existing plans. We have asked each site to report the number of patients with: no individual pain plans, IPP's written in a clinic note, IPP's accessible somewhere else in the EHR that is easily accessible.

6. **What about patients who have restrictive pain plans (one that limit opioid exposure due to concerns over the risks of opioids v benefits for the individual patients? That might significantly influence the subjects' satisfaction with the quality of their pain management. Will those patients be included in this study?**

We can add "patients with existing IPP's that do not allow opioid administration" as an additional exclusion factor.

NHLBI / SCD-SCDID OSMB Protocol Review Form, Reviewer 4, Nov 26.
**Sickle Cell Disease Implementation Consortium**<br>**Implementing an Individualized Pain Plan (IPP) with Patient and Provider Electronic Health Record Access, for Adult Emergency Department Treatment of Vaso-occlusive Episodes in Sickle Cell Disease: A Pre-post Study Design**

Instructions: This form is designed to guide protocol reviews and to collect comments, issues of concern, requests for additional information or clarification, and to record an initial approval recommendation. Please forward the completed form to [redacted] y Tuesday, November 19, 2019

Study Objectives and Feasibility	Acceptable	Needs Discussion	Not Reviewed
Adequacy of previous research and/or clinical studies of proposed treatment or therapy	X		
Scientific merit of the study objectives; primary and secondary endpoints	X		
Interventions: treatment or therapeutic regimen	X		
Data to be collected: laboratory tests, clinical exams, outcome(s) of interest	X		
Feasibility: including recruitment strategies, target recruitment goals, and timetable for study completion	X		

COMMENTS:

1. **Second paragraph prior to section 2.2, sentence spanning the two pages "By assessing use of the IPP, we will be able to compare patient and provider outcomes, statistically, …" It is not clear how you will do this. Are you referring to tables 2 and 3 here and allowing X in your LMM equation to be a specific implementation component (likely the optional, as the required are going to be confounded with each other)? Please elaborate.** *Deleted*

Study Design and Statistical Approach	Acceptable	Needs Discussion	Not Reviewed
Study hypothesis(es)		X	
Experimental design: sample size, use of controls or standard treatment or therapy, single/multicenter design		X	
Statistical approach, power and data analysis plan		X	

COMMENTS:

1. **Paragraph prior to section 2.2 Using the Reach Effectiveness Adoption, Implementation and Maintenance (RE-AIM) for planning and Evaluation. "As a preliminary attempt …" This sounds as if you are interested in effect modification. This is also hinted at in section 2.5 "readiness assessment". I don't see items for subgroup analyses or effect modification in the analysis plan**. *Exploratory because we won't have enough power for this.*

2. **I agree that a pre-post design is appropriate for this study. Would it be helpful to have a "registry" of sites that do not implement the protocol and collect similar outcomes on participants to see if there is an independent time effect?**

All sites are encouraged to participate in this project, thus we do not have this option, although it would be helpful.

3. **If the patient visits a non-participating ED, will their experiences still be collected, almost as a control? I suppose it depends on how many non-participating ED visits will occur.**
   While desirable, we do not have the resources to collect data at other EDs.

4. **Is the difference of SD clinically meaningful? If you are averaging three questions together, a change of an outcome of 0.39 is essentially a one-unit increase on one of the questions. It's good to be able to power something that small, but I would hope a more clinically meaningful difference is defined.** *Addressed in other response.*

5. **Is there any expectation that the impact of the satisfaction of the IPP would vary according to how many visits there were to the ED for the participant.** *It is possible. Not enough power for the analysis. We will take the first visit of the patient in the stimulation.*

6. **Section 3.2. Will you be measuring /following adherence to follow-up questionnaires?**
   *We will measure missing data for the follow up surveys that are distributed after an ED visit***. Will you use all data (to get better baseline estimates) or just participants who have a follow-up ED visit?** *We will use all data at baseline for baseline sample descriptions, but we will only be able to assess implementation and intervention outcome based on participants who have an ED visit for VOE during the implementation period.*

7. **Qualitative Data, Do sites have analysis teams? What is their membership? How many people per team? How are they trained?** *All analysis is done at each site, will be members of the research team, and will be trained by the RTI team.*

Study Operations	Acceptable	Needs Discussion	Not Reviewed
Plans for study operations: organization of clinical centers, governing committees, data coordinating center	X		
Plans for training clinical center personnel: good clinical practice, research goals, protocol details and implementation, and study operations	X		
Plans for data acquisition: data management, quality assurance, and ultimate transfer of data to NHLBI		X	

COMMENTS:

1. **How will you prioritize the 5 provider and 5 patient interviews as you move forward in real time, within 2 weeks of the visit? Who will make that decision? If it is based on IPP use, then are you targeting mostly those who use the IPP, who don't use the IPP or a mix of both? Will this be with the aid of the DCC? If it is related to the change in the primary outcome, will that cause any conflict for the site to see change in outcome data (I know it isn't randomized)**
   We will recruit based on IPP use and target a mix of both. Sites will make recruitment decisions on their own, but we will have regular biweekly meetings to debrief on the recruitment process.

2. **Prior to tracking and reporting implementation activities and strategies. I'm a bit confused as to the difference between provider and participant surveys and the ancillary study described in Appendix A.Please clarify.**
   The patient and provider survey and interview is to assess implementation and intervention outcomes. The parallel study is to track and report implementation strategies and their specifications used by each site, which will include interviews of site PI or research staff.

3. ***How many visits per participant do you expect?*** *1.* ***Is the ED visit a study visit?*** No

4. **Provider participants - If you are not getting written consent for the surveys, will you be able to link the pre-post surveys together (or the survey of the provider at the beginning of the study to the post-SCD participant ED contact survey)?**
   Yes we will establish a participant ID for providers.

5. **Please help me understand why the time of the first opioid administration is important. Is it a measure of how quickly the IPP can be implemented?**
   This is not directly related to the protocol but important information.

6. **RE-AIMTable . I'm confused by this one. Aren't written IPPs within the EHR the definition of "adopted" for the IPP? What does "after study targets are met" mean?**
   1) We define adoption at the systematic level. Since IPPs are supposed to be written for all participants, whether 100% of the participants had those written are an implementation fidelity issue, hence we categorize it in the implementation section of the RE-AIM model (same with provider use, since all of them are supposed to use the IPPs, whether they use it or not is defined as an implementation fidelity assessment rather than an adoption assessment). 2) Each site has a target of enrolling 40 participants, but sites can keep enrolling, or providing IPPs to more patients with SCD beyond the 40 participant's target.

Human Subjects Protection	Acceptable	Needs Discussion	Not Reviewed
Model informed consent document	x		
Data and safety monitoring plan: including adverse event notification, medical monitor, and stopping guidelines			X
Subject population: including appropriate representation of minorities, women, and children	X		
Discussion of Equipoise			X

1. Instrument B. I4. Please be more gender inclusive. Revised

Approval Recommendation	Approve	Approve with Changes*	Do Not Approve	Postpone Approval Recommendation Until After Discussion
Protocol				X
Model Consent Document		X		
Other materials				X

Minor Comments:

2. For aim 2. You don't include the "effect" of the RE-AIM acronym. Any reason why? Because the Aim1 & sub-aims are all about 'effect', the second paragraphs are for the rest of the RE-AIM.
3. Appendix .
    a. A few of the "level of measurement" is system. Does that refer to the Site/ED? Yes, the variable is not measured from the patient or provider, but system/ED EHR data.

Date: Jan 9, 2019

To: OSMB Members, SCDIC U01, NHLBI

RE: Response to OSMB Protocol approval meeting December 2, 2019

From: Allison King and Paula Tanabe, ED Intervention, Co-chairs

OSMB Concern Primary outcome measure: *perceived quality ED pain treatment*.

Measure items:
1)How satisfied were you with the care you received?

2)How much were the emergency department doctors and nurses able to help your pain?

3)How much did the emergency department doctors and nurses believe that you had very bad sickle cell pain?

Specific OSMB concerns:

1. **Construct Validity of the primary outcome measure**
2. **Reason of choosing a patient satisfaction measurement as our primary outcome**
3. **Temporality** (baseline measurement requests the patient to provide an average of the quality of their ED pain treatment for the last 12 months; comparison measure for future ED visits, and reported within 72 hours of the ED visit)

Study Team Response

1. **Construct Validity of the primary outcome measure [Protocol revised]**

Our primary aim is to assess patient's satisfaction of ED pain treatment through their perceived quality of ED pain treatment. A recent systematic review published in 2018 identified 24 instruments used between 1997 and 2017 assessing patient reported outcomes (PRO) in populations with SCD (both adults and children) in the U.S.[1] Five of the 24 instruments were developed for adult SCD patients**.**[1] **The Adult Sickle Cell Quality of Life Measure (ASCQ-Me) Quality of Care (QOC) measure was the only one that assesses treatment satisfaction.**[1] It was rated as having both **good content validity and internal reliability**.[1] Unfortunately, none of the 24 PRO instruments provided information regarding threshold of minimally important change.[1] With its limitation in mind, the workgroup decides that ASCQ-Me QOC is the best available instrument to use for the study population and our primary aim.

The ASCQ-Me QOC measure was modeled after the Consumer Assessment of Healthcare Providers and Systems (CAHPS) surveys, a widely used tool by the Centers of Medicare and Medicaid Services (CMS), Department of Defense and Department of Veterans Affairs.[2-4] **There is evidence of the reliability and validity of the instrument and the ASCQ-Me QOC measure has been used by other research studies.**[3,5]

The ASCQ-ME QOC includes global evaluation of care for SCD received from regular provider and **the ED**, and QOC in three domains: provider communication, **ED care**, and access.[3] To avoid participant survey fatigue and ensure we collect information that is most helpful and relevant, the workgroup chose the three questions from the ASCQ-ME QOC to measure perceived quality of ED pain treatment. **Question 1) is to address global evaluation of care for SCD in the ED. Question 2) and 3) is to assess QOC-ED care.**

Within the timeframe of the SCDIC grant, we do not have time to conduct a rigorous psychometric testing of the items prior to initiation of the project. However, we believe the ASCQ-Me QOC validation that was conducted is sufficient as it identified the construct of “quality of care” in ED, and the “care” we are providing is pain management. As no instrument has provided a threshold of minimally important change, after numerous discussions, the workgroup has reached the conclusion that a 0.5 SD change will be a significant change.

2. Reason of choosing a patient satisfaction measurement as our primary outcome[Protocol revised]

Over the past 18 months, the workgroup has explored possibilities of using other patient outcomes as our primary outcome, but eventually we decided that they were not as feasible or not as reliable compared to assessing patient satisfaction, which is also an important patient outcome. The table below suggests other possible measures of pain management and rationale as to why they were not selected as the primary outcome.

Measure	Reason of not choosing as the primary outcome
Visual analogue scale (0-10) – prospectively obtained from patient during treatment for pain. (gold standard)	Sites are not equipped to obtain pain ratings during the ED visit. EDs typically have only 1-3 VOE visits/day. Given the other expectations of the SCDIC grant (ongoing registry data collection and participation in a 2nd and possible 3rd implementation science project), it is not possible to have research staff available 24/7 to obtain this data. Requiring ED providers to have that rating will add significant implementation fidelity challenges and is not feasible.
Visual analogue scale (0-10) – documented in the EHR	There is tremendous variability as to “if and when” pain scores are documented. Reliant on the EHR would result in a very large amount of missing data for the primary outcome.
Hospital admission	A proxy measure of the effectiveness of pain management. We originally proposed this as the primary outcome, however the sample size was over 100 subjects/site. When proposed to the sites, all 8 sites stated they would not be able to participate in the project as it was not feasible to enroll 100 participants at each site.

The workgroup values assessing implementation outcome. Table 4 of the protocol has listed many implementation outcomes that we will measure. However, the workgroup aims to demonstrate that the IPP has a positive impact on patient care. Having a PRO instead of implementation outcomes as our primary outcome is the best approach.

3. Temporality of measure (validity threat) [Protocol revised]

We acknowledge that there was a concern of recall bias at baseline, and comparing past 12 months satisfaction at baseline and last visit satisfaction at follow-up. To address these concerns, we propose a change in our **inclusion criteria**: we will recruit individuals with SCD who have a participating site ED VOE visit **within the past 90 days.** We will also change our **baseline assessment** to perceived quality of care **of the last ED visit (happened within 90 days),** instead of for the past 12 months. With this revision, we will be able to compare baseline last visit satisfaction with follow-up last visit satisfaction. This change is summarized in the table below.

We may need additional recruitment strategies. If recruiting via phone, the team needs to schedule an orientation, during which the team member will help patients install patient portal app on the phone, show them how to access the IPP, and let patients show the staff how to access (a teach-back method). Some patients who have a recent ED VOE visit will have a scheduled clinic visit within a few weeks that could serve as the orientation. The research teams will make efforts in increasing the show up rate of the orientation. Study team can also screen patients who have scheduled clinic visits and recruit those who have an ED VOE visit within 90 days at the clinic.

We propose to set the eligibility criteria of having an ED VOE visit within the last 90 days, instead of 30 days, based on work group discussions and our registry data. Using registry data from one of the study site, Washington University in St. Louis, as an example: the site maximized recruitment efforts and approached all eligible patients with a clinic visit from Sep 2017 to Jan 2019 (18 months), enrolled 271 patients from clinic visits (decline rate less than 3%). At the time of

enrollment, 35 (12.9%) had an ED VOE visit within 30 days, and 105 (38.7%) had an ED VOE visit within 90 days. This is for a 18 months enrollment period, so for this proposed study which has a much shorter enrollment period, setting the eligibility criteria of having an ED VOE visit within the last 30 days will make it impossible for sites to reach enrollment target (e.g. Washington University will be able to recruit less than 15 patients over a 6 months enrollment period from the Clinic). After OSMB reviews, sites are expected to have additional recruitment strategies, however, recruiting from the clinic or from the registry will be the most effective recruitment strategies (recruiting from the ED, many individuals will not be eligible if they did not have a participating site clinic visit within the past 12 months and/or the hematologist is not able to build a pain plan for them). So we decided to set the eligibility criteria to having an ED VOE visit at participating EDs within the last 90 days, instead of 30 days.

Additional major changes to the protocol:

1. Changed follow-up time window from 72 hours to 96 hours to give research staff time to follow up with patients with an ED VOE visit on Friday.
2. Added a new criteria for the qualified ED visit: the first participating ED VOE visit of the month (p.17). The rationale of this criterion is to alleviate the burden of data collection and retrieval if the patient has more than one visit in a month.
3. Revised site characteristics table regarding sites' existing patient and provider IPP access (p. 12)
4. Revised statistical analysis section (p.25)
5. Added a patient tracking log to appendix
6. Revised instruments and timeline based on the proposed changes
7. Revised patient consent form

	Previous design	Revision
**Primary Outcome**	Perceived quality of ED pain treatment (Three questions from the ASCQ-Me QOC)	Perceived quality of ED pain treatment (Three questions from the ASCQ-Me QOC)
**Inclusion criteria**	• Confirmed SCD diagnosis.<br>• English speaking<br>• Age 18–45 years<br>• Access to a cellular/mobile smart phone with unlimited data plan and free text messages (either Android or iPhone are acceptable)<br>• At least one VOE visit to the participating site's ED **in the past 12 months**<br>• At least one visit at the study site SCD clinic within the past 12 months<br>• Willing and cognitively able to give informed consent	• Confirmed SCD diagnosis.<br>• English speaking<br>• Age 18–45 years<br>• Access to a cellular/mobile smart phone with unlimited data plan and free text messages (either Android or iPhone are acceptable)<br>• At least one VOE visit to the participating site's ED **in the past 90 days**<br>• At least one visit at the study site SCD clinic within the past 12 months<br>• Willing and cognitively able to give informed consent
**Recruitment and Enroll**	• Ongoing recruitment<br>• Consent and enrollment during a clinic visit	• Ongoing recruitment<br>• Review EHR and identify patients meeting inclusion criteria.<br>Suggest comparing these patients to a list of enrolled patients in the registry to decrease burden of cold call contact and IRB.<br>• Contact patient by phone, obtain verbal consent to participate OR consent and enrollment during a clinic visit
**Baseline Patient Survey**	**One Survey**<br>**Demographics & Primary outcome assessment 3 questions**<br>• Administered at enrollment in person<br>• Perceived quality of ED pain treatment over the **past 12 months**<br>**Pain plan related assessment**<br>• Administered at enrollment in person	**Two Parts Survey**<br>**Demographics & Primary outcome assessment 3 questions**<br>• Administered at enrollment via phone<br>• Perceived quality of ED pain treatment of **the last ED visit** (which occurred no > 90 days prior)<br>**Pain plan related assessment**<br>Administered at **orientation** (after training session)<br>Or<br>**One Survey** (if orientation is within 90 days)
**Building pain plan and training session**	• Request hematologist to write/update individual pain plan (IPP)<br>• Obtain written consent and review IPP with patient in person, etc. at **enrollment**	• Request hematologist to write/update individual pain plan (IPP)<br>• **orientation**: Schedule a research study visit, or clinic visit scheduled as the orientation<br>• Obtain written consent and review IPP with patient in person, etc. during **orientation**

References

1. Sarri G, Bhor M, Abogunrin S, et al. Systematic literature review and assessment of patient-reported outcome instruments in sickle cell disease. *Health and quality of life outcomes.* 2018;16(1):99.
2. Health NIo. ASCQ-Me Quality of Care (QOC) Measure. n.d.; http://www.ascq-me.org/Measures/Quality-of-Care-QOC-Measure.
3. Evensen CT, Treadwell MJ, Keller S, et al. Quality of care in sickle cell disease: Cross-sectional study and development of a measure for adults reporting on ambulatory and emergency department care. *Medicine.* 2016;95(35).
4. Treadwell MJ, Hassell K, Levine R, Keller S. Adult sickle cell quality-of-life measurement information system (ASCQ-Me): conceptual model based on review of the literature and formative research. *The Clinical journal of pain.* 2014;30(10):902.
5. Smith WR, k McClish D, Lottenberg R, et al. Comparison of Adult and Adolescent Quality of Ambulatory and Emergency Care in Sickle Cell Disease: Ascq-Me and the SHIP-HU Study. Am Soc Hematology; 2017.

NHLBI / SCD-SCDID OSMB Protocol Review Form, Feb 2020
**Sickle Cell Disease Implementation Consortium**<br>**Implementing an Individualized Pain Plan (IPP) with Patient and Provider Electronic Health Record Access, for Adult Emergency Department Treatment of Vaso-occlusive Episodes in Sickle Cell Disease: A Pre-post Study Design (version 1.1)**

Instructions: This form is designed to guide protocol reviews and to collect comments, issues of concern, requests for additional information or clarification, and to record an initial approval recommendation. Please forward the completed form to [redacted] y February 17, 2020

Study Objectives and Feasibility	Acceptable	Needs Discussion	Not Reviewed
Adequacy of previous research and/or clinical studies of proposed treatment or therapy			
Scientific merit of the study objectives; primary and secondary endpoints			
Interventions: treatment or therapeutic regimen			
Data to be collected: laboratory tests, clinical exams, outcome(s) of interest			
Feasibility: including recruitment strategies, target recruitment goals, and timetable for study completion			

COMMENTS:

1. **Can the investigators clarify where they are collecting data on whether what the participant received in an ED visit was consistent with the IPP (I may have missed it but it doesn't look like it is in the data extracted from the ED visits). I see the providers are asked about whether they followed the IPP but collecting the objective data that the plan was followed would be important. What if a provider says they followed it but in fact it was not followed. The charts are being extracted anyway so would be fairly easy to collect.**
   This information will be extracted in the EHR retrieval data. Instrument B "Patient Information Form: RESARCH TEAM" "End of implementation EHR retrieval data (12 months after enrollment, VOE visits, enrolled patients) - #6 "Pain management data, first dose – a. Drug name, b. Drug dose, c. Drug unit, d. Drug route"

2. **For data analysis plan. I believe you are trying to show a three-level mixed effects model. Your random intercept includes the overall mean (\mu), the random site effect (\alpha_j) and the nested individual in site effect (\nu_{ji}). However, I do not agree with the parameterization of your slope. \Beta X is the fixed slope. The random slope should be \tau_j X. I do not believe that the random effect for site should equal the random effect for the slope (hence the \alpha_j on the slope should be \tau_j). I also do not believe you need the \beta with the \tau_j X term. Then the individual error is \epsilon_{jti}. "because the site is a random effect, the interaction is also a random effect". I do not believe that the slope needs to be random because the intercept is random. It can be that way, but it doesn't statistically need to be that way.**
   Quantitative data analysis plans have been updated please see track changes.

Study Design and Statistical Approach	Acceptable	Needs Discussion	Not Reviewed
Study hypothesis(es)			
Experimental design: sample size, use of controls or standard treatment or therapy, single/multicenter design			
Statistical approach, power and data analysis plan			

1. **Regarding the "patient eligibility criteria #1, diagnosis. If no medical record is available are the sites required to conduct their tests as confirmation, or is it optional?**
If no medical record is available the enrolling site will conduct a laboratory test as confirmation – protocol text updated

2. **For primary outcome "… will be measured with three questions, using their composite scores". By composite do you mean the sum of their scores?**
A composite score is calculated as the average of the three responses

3. **For the qualitative evaluation, it indicates that "… we will elaborate on the RE-AIM quantitative findings with a brief interview for a small number of patients and ED providers." Please provide an idea of a small number (at least 5 of each according to paragraph 2 below this one). Will interviews only include participants who had a follow-up ED visit?**
Yes, each participating Center will conduct at least five provider and five patient interviews with participants who had an ED VOE visit post enrollment.

4. **In the response the investigators report that they will enroll people with VOC within 30 days of enrollment but the protocol says within 90 days. If feasible it would be better to limit recall to the last 30 days.**
The protocol is correct at 90 days. Due to small patient population, 30 days would not provide a reasonable potential participant poll to all investigators to meet enrollment goals.

5. **Investigators should consider excluding participants who already have IPP that may have been used at the visit prior to enrollment..**
While the study sites will track who already has an IPP (prior to enrollment) and who does not, excluding those with an existing IPP would be detrimental to enrollment at some sites. Please note, having a pre-enrollment IPP in place does not imply the patient has had an ED visit with that IPP in place or that it was accessible to the patient and their ED care provider during any pre-enrollment ED visit

Study Operations	Acceptable	Needs Discussion	Not Reviewed
Plans for study operations: organization of clinical centers, governing committees, data coordinating center			
Plans for training clinical center personnel: good clinical practice, research goals, protocol details and implementation, and study operations			
Plans for data acquisition: data management, quality assurance, and ultimate transfer of data to NHLBI			

COMMENTS:

1. **For recruitment (page 12 "required patient-related strategies") the first bullet is "enrollment of patients in clinic or hospital." I assume that you would only enroll patients if they had an ED visit within the last 90 days. Are you requiring the last ED visit to be at one of the participating ED's, or could it have been at an external ED? If you are allowing it to be an external ED, are you capturing this information?**
   ED visits must be at participating EDs. See Eligibility Criteria - Patient Participants bullet 5 "At least one VOE visit to the participating site's ED in the past 90 days from enrollment"

2. **Page 16, interventions two paragraph above "implementation strategies", "The hematologist or SCD provider will review… The IPP will be reviewed every 6 months by the SCD provider and updated as needed." Given there is a 1-year follow-up, then it would only need to be updated once in the study, at 6 months, correct?** *Correct. Updated to say "6 months post-enrollment"*

Human Subjects Protection	Acceptable	Needs Discussion	Not Reviewed
Model informed consent document			
Data and safety monitoring plan: including adverse event notification, medical monitor, and stopping guidelines			
Subject population: including appropriate representation of minorities, women, and children			
Discussion of Equipoise			

Approval Recommendation	Approve	Approve with Changes*	Do Not Approve	Postpone Approval Recommendation Until After Discussion
Protocol				
Model Consent Document				
Other materials				

***Please describe the changes deemed necessary for approval:**

1. **The inclusion criteria "access to a cellular/mobile smart phone with unlimited data plan and free text messages (87)" seems unnecessarily restrictive. While I could not find statistics on what percentage of smart phone users have unlimited data plans, I would image that it would be low. If this criterion was eliminated, the potential "cost" of participation would need to be included in the informed consent form.**
   removed the requirement for an unlimited data plan and free text messaging – new sentence reads "access to a cellular/mobile smart phone with access to text messaging" We added the potential costs incurred to the patient if they do not have unlimited data/free text messaging to the content form.
